# Serum NMR metabolomics to differentiate haematologic malignancies

**DOI:** 10.18632/oncotarget.25311

**Published:** 2018-05-11

**Authors:** Wojciech Wojtowicz, Angelika Chachaj, Andrzej Olczak, Adam Ząbek, Elżbieta Piątkowska, Justyna Rybka, Aleksandra Butrym, Monika Biedroń, Grzegorz Mazur, Tomasz Wróbel, Andrzej Szuba, Piotr Młynarz

**Affiliations:** ^1^ Wroclaw University of Technology, Department of Bioorganic Chemistry, Wroclaw, Poland; ^2^ Wroclaw Medical University, Department of Angiology, Wroclaw, Poland; ^3^ Opole University of Technology, Faculty of Electrical Engineering, Automatic Control and Informatics, Opole, Poland; ^4^ Wrocław Research Centre EIT+, Wroclaw, Poland; ^5^ Department of Haematology, Blood Neoplasms, and Bone Marrow Transplantation, Wroclaw Medical University, Wroclaw, Poland; ^6^ Department of Internal Medicine, Wroclaw Medical University, Wroclaw, Poland; ^7^ Department of Physiology, Wroclaw Medical University, Wroclaw, Poland

**Keywords:** metabolomics, haematological malignancies, nHL, AML, CLL

## Abstract

Haematological malignancies are a frequently diagnosed group of neoplasms and a significant cause of cancer deaths. The successful treatment of these diseases relies on early and accurate detection. Specific small molecular compounds released by malignant cells and the simultaneous response by the organism towards the pathological state may serve as diagnostic/prognostic biomarkers or as a tool with relevance for cancer therapy management. To identify the most important metabolites required for differentiation, an ^1^H NMR metabolomics approach was applied to selected haematological malignancies. This study utilized 116 methanol serum extract samples from AML (n= 38), nHL (n= 26), CLL (n= 21) and HC (n= 31). Multivariate and univariate data analyses were performed to identify the most abundant changes among the studied groups. Complex and detailed VIP-PLS-DA models were calculated to highlight possible changes in terms of biochemical pathways and discrimination ability. Chemometric model prediction properties were validated by receiver operating characteristic (ROC) curves and statistical analysis. Two sets of eight important metabolites in HC/AML/CLL/nHL comparisons and five in AML/CLL/nHL comparisons were selected to form complex models to represent the most significant changes that occurred.

## INTRODUCTION

Haematological malignancies are clonal diseases. Neoplasms of the haematopoietic system are derived from myeloid lineage stem or progenitor cells, while the tumours of the lymphatic system originate from lymphoid lineage precursor or mature cells [1]. The factors causing these malignant transformation disorders are both genetic and environmental. They lead to abnormal signal transduction and gene expression in the cell and to disturbances during key haematopoiesis processes, such as self-renewal, proliferation and differentiation [1]. According to the Polish National Cancer Registry, the number of new cases of haematological malignancies increased near 2-fold in the past three decades. In 1990, the incidence rate was estimated as 8.8 / 100000 (10.4 for men and 7.4 for women), and in 2010 it was 16.8 / 100 000 inhabitants (18.1 for men and 15.5 for women). An important risk factor for haematological disorder is the patient's age. The one of the most frequent haematological disorders, which occur in adults, are acute myeloid leukaemia (AML) in the haematopoietic system, non-Hodgkin's lymphoma (nHL) and chronic lymphocytic leukaemia (CLL) among lymphatic system neoplasms. Most cases (approximately 60%) are recorded between 50 and 79 years of age [2, 3, 4]. The 5-year survival rates among these haematological malignancies are 27% for AML, 70% for nHL and 83% for CLL patients [1, 3, 4]. The early diagnosis and effective treatment of haematological malignancies continues to be an overwhelming challenge. Malignant cells exhibit a distinct metabolic phenotype, which may be reflected by the release of low molecular weight compounds [5, 6]. The analysis of changes in these compounds may serve as a diagnostic/prognostic biomarkers not only for the malignancies but also as a tool with relevance to cancer management, therapy and monitoring [7]. Recently, numerous studies have characterized the metabolic profiles of a variety of malignant tumors, including brain, lung, prostate, pancreatic, breast, ovarian, liver and thyroid [8, 9].

The purpose of the study was to determine the metabolic profiles of the three common haematological malignancies in adults, acute myeloid leukaemia (AML), non-Hodgkin's lymphoma (nHL) and chronic lymphocytic leukaemia (CLL), using an metabolomics approach, for highlighting potential important influence on organism metabolism caused by haematological malignancies and indication possible biomarkers.

## RESULTS

**Figure 1:**
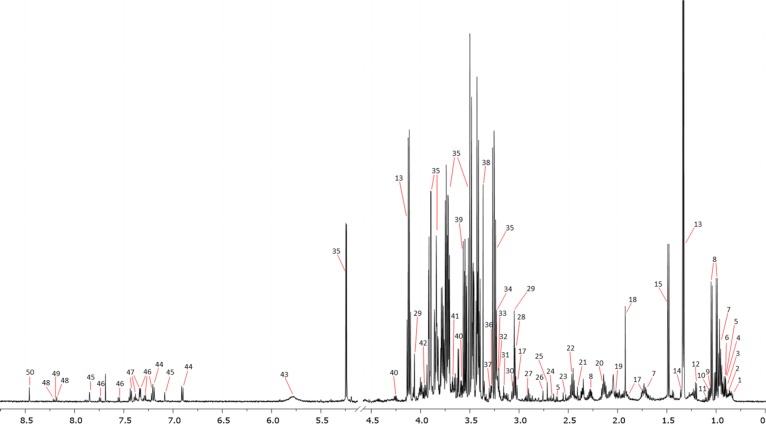
Representative ^1^H NMR spectrum with marked resonance signals 1 - 2-Hydroxyisovalerate, 2 - 2-Hydroxybutyrate, 3 - Isovalerate, 4 - Isoleucine, 5 - 2-Oxoisocaproate, 6 - Alloisoleucine, 7 - Leucine, 8 - Valine, 9 - Isobutyrate, 10 - 2-Methylglutarate, 11 - 3-Methyl-2-oxovalerate, 12 - 3-Hydroxybutyrate, 13 - Lactate, 14 - 2-Hydroxyisobutyrate, 15 - Alanine, 17 - Lysine, 18 - Acetate, 19 - Proline, 20 - Glutamine, 21 - Succinate, 22 - Glutamate, 23 - Citrate, 24 - Methionine, 25 - Dimethylamine, 26 - Sarcosine, 27 - N,N-Dimethylglycine, 28 - Creatine, 29 - Creatinine, 30- Ornithine, 31 - Dimethyl sulfone, 32 - Choline, 33 - *O*-Phosphocholine, 34 - sn-Glycero-3-phosphocholine, 35 - Glucose, 36 - Betaine, 37 - Taurine, 38 - Methanol, 39 - Glycine, 40 - Threonine, 41 - Glycerol, 42 - Serine, 43 - Urea, 44 - Tyrosine, 45 - Histidine, 46 - Tryptophan, 47 - Phenylalanine, 48 - Hypoxanthine, 49 - Oxypurinol, and 50 - Formate.

Altogether, 50 metabolites were assigned (Figure 1 and Supplementary Table 1) and 18 resonance signals were marked as unknown. From the identified metabolites, 45 and the 18 unknown signals were used for the chemometric models due to their non-overlapping signals. The found metabolites exhibited different trends in the particular comparisons, such as only increasing, only decreasing and with variate trends (Supplementary Table 2).

The obtained chemometric models, which were based on VIP-PLS-DA, could classify patients’ haematological malignancy samples into individual groups versus the healthy control group. Comparisons between the individual haematologic malignancies were also based on VIP-PLS-DA models (calculated separately) and showed lower model parameter values than comparisons with healthy individuals (Table 1). In general, five of the eight models passed the validation test, though most of these models did not include CLL.

**Table 1:** The VIP-PLS-DA model parameters for each comparison based on serum samples

Comparison	Latent variables	R^2^X(cum)	R^2^Y(cum)	Q^2^(cum)	CV-ANOVA	AUC training	AUC test
**HC/AML/CLL/nHL**	2	0.370	0.267	0.223	3.96E-12	-	-
**HC /AML**	2	0.512	0.864	0.744	3.18E-05	1.000	0.975
**HC/CLL**	2	0.472	0.662	0.248	1.05E-01	0.852	0.588
**HC/nHL**	2	0.614	0.622	0.37	1.08E-02	0.898	0.853
**AML/CLL/nHL**	2	0.366	0.394	0.315	4.98E-11	-	-
**AML/nHL**	2	0.530	0.692	0.575	7.74E-04	0.929	0.837
**AML/CLL**	2	0.459	0.719	0.383	6.20E-02	0.879	0.932
**CLL/nHL**	2	0.582	0.469	0.287	7.84E-02	0.837	0.655

### Analysis of all the studied groups (AML, CLL, nHL, and HC)

**Figure 2:**
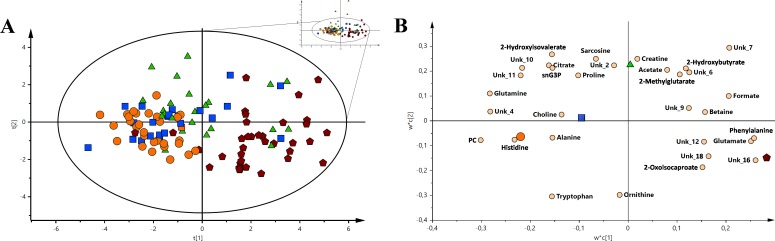
The VIP-PLS-DA models for all the groups used in the study For a better representation, only Hotelling's T2 range is shown. **(A)** The PLS-DA general model with all the sample groups. **(B)** Loading plot of the VIP-PLS-DA model for all groups and samples used in the study. Red pentagon – AML; Orange circle – HC; Green triangle – nHL and Blue box – CLL. The inserted reduced figure serves to show outliers related to the variability in the studied groups.

The analysis of the VIP-PLS-DA models calculated for three malignancies units and control group using a set of metabolites with the greatest discriminatory potential (Figure 2A and 2B). This pool of metabolites included (sequentially decreasing VIP values) *O*-phosphocholine, glutamine, phenylalanine, tryptophan, glutamate, histidine, formate, 2-hydroxyisovalerate, citrate, sn-glycero-3-phosphocholine, ornithine, 2-oxoisocaproate, 2-hydroxybutyrate, alanine, 2-methylglutarate, betaine, sarcosine, creatine, acetate, proline, and choline.

**Figure 3:**
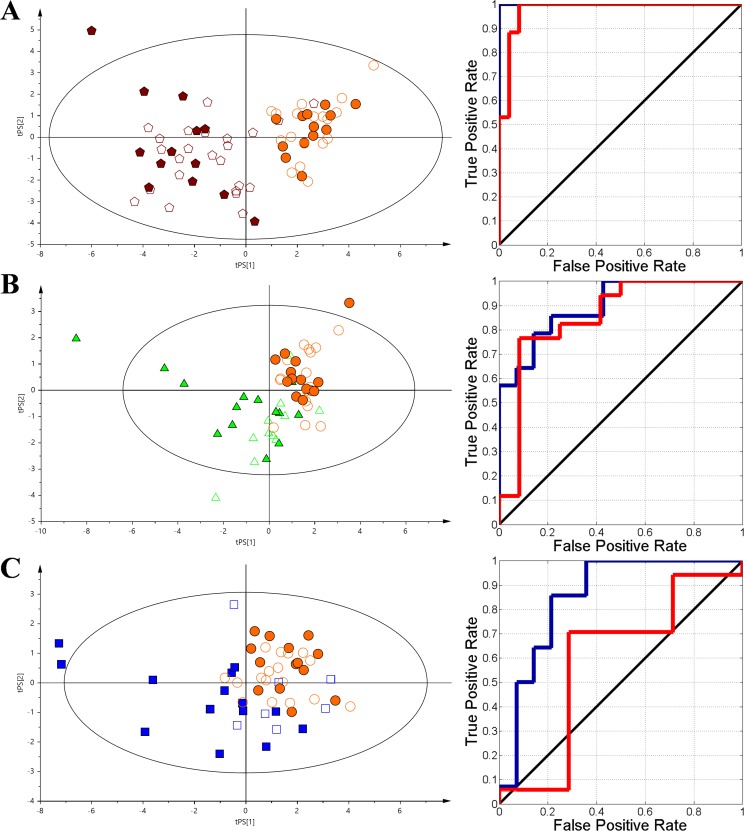
The VIP-PLS-DA models plots with the training (no fill) and test (fill) set samples and ROC curves The dark blue curve represents the training set and the red curve represents the test set. **(A)** - HC vs AML; **(B)** - HC vs nHL, **(C)** - HC vs CLL. Red pentagon – AML; Orange circle – HC; Green triangle – nHL and Blue box – CLL.

The loading plot (Figure 2B) of comprehensive model allows for metabolite selections and verification, which had the most important contribution to the differentiation between investigated groups. From the determined metabolites, three of the most important for differentiation among the identified and unidentified compounds based on the closest distance to Y-variable have been selected for the HC vs. AML vs. CLL vs. nHL comparison (R^2^X = 0.401, Q^2^ = 0.218). Hence, the following sets of identified compounds were chosen: (1) for HC – histidine, *O*-phosphocholine, and glutamine; (2) for AML – phenylalanine, glutamate and formate; (3) for CLL – choline, alanine, and proline and (4) for nHL– creatine, sarcosine, and acetate. Among the unidentified metabolites, unk_4 was important for HC, unk_16 for AML, and unk_2 for nHL, while no unidentified metabolites were important for CLL. Moreover, using a single metabolite in multiple groups was avoided. The introduced general comparisons shown in Figure 2A and 2B demonstrate the ability to distinguish between the investigated groups, though it does not accurately reveal the metabolite variation between the specific particular comparisons, e.g., HC vs. AML. Therefore, more detailed chemometric models based on comparisons between two groups were calculated (Table 1 and Figures 3 and 6). The best classification was achieved for the comparison between the HC and AML groups. This model obtained the greatest ROC curve (AUC_training_ 1.00; AUC_test_ 0.975) and basic parameter values among the models (Table 1 and Figures 3A and 3B). In contrast, assessment of HC and CLL groups showed the lowest model parameters values (AUC_training_ 0.852; AUC_test_ 0.588) among models based on the HC vs. haematologic malignancy group comparisons. However, the separation between the selected groups was still observed.

A set of 10 identified metabolites were determined for the HC vs. AML model (R^2^X = 0.512, Q^2^ = 0.744) (Figure 3A) - 2-hydroxybutyrate, 2-methylglutarate, 3-methyl-2-oxovalerate, betaine, formate, glutamate, *O*-phosphocholine, phenylalanine, tryptophan, and histidine were statistically significant and had major contributions to the differentiation based on their VIP values (Supplementary Table 2). Considering the same steps as in the complex model (Figure 2A and 2B), nine relative integrals from unspecified resonances signals, including unk_3, unk_4, unk_6, unk_7, unk_9, unk_10, unk_11, unk_12, and unk_16, were also selected. In the HC vs. nHL discrimination model (R^2^X = 0.614, Q^2^ = 0.37) (Figure 3B), four of the resonances were determined within the relative integral values. Two of the resonances were identified as formate and *O*-phosphocholine and the two unassigned specific metabolites were marked as unk_4 and unk_7. In the midst of all the studied haematological malignancies, the CLL group showed the lowest separation vs. the HC group (R^2^X = 0.472, Q^2^ = 0.248) (Figure 3C) based on the VIP-PLS-DA model. Despite of low separation, 4 identified metabolites and 4 unknowns were determined, including dimethylamine, formate, glutamate, N,N-dimethylglycine, unk_3, unk_6, unk_7, and unk_9, which significantly affected the discrimination between the investigated groups.

**Figure 4:**
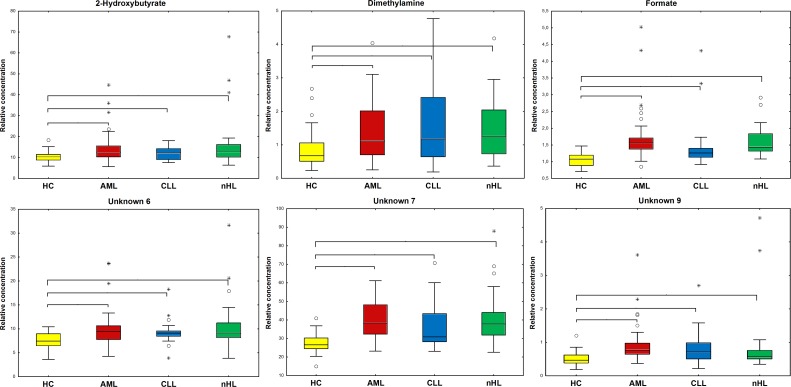
Boxplots of the statistically important metabolites (*p*<0.05) between HC and the haematological cancers Braces mark the comparisons where the selected metabolites are statistically important. Whiskers - non-outlier min-max range; ^*^ - extreme; ° - outlier; bar - median; box - Q1-Q3 interquartile range; yellow – HC; red – AML; blue - CLL; and green - nHL.

Statistical analysis performed for all found compounds based on median or mean levels showed that six metabolites were statistically significant (M-W or *t*-test) in each of three comparisons between HC and the haematological malignancies (Figure 4).

### Analysis of the haematological malignancy groups (AML, CLL, and nHL)

**Figure 5:**
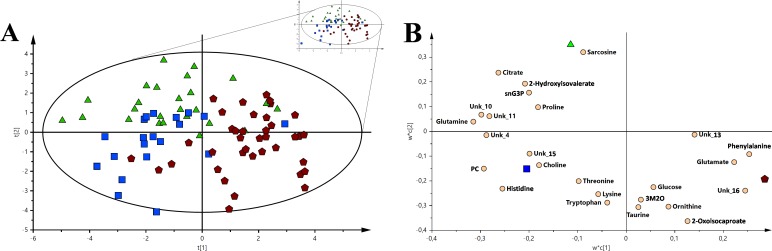
The VIP-PLS-DA models for all the groups used in study For a better representation, only Hotelling's T2 range is shown. **(A)** The VIP-PLS-DA model with the haematological malignancy groups. **(B)** VIP-PLS-DA loading plot for the haematological malignancy groups. Red pentagon – AML; Green triangle – nHL and Blue box – CLL. The inserted reduced figure serves to show outliers related to the variability in the studied groups.

The complex comparisons between the studied haematological cancers were based on a chemometric VIP-PLS-DA model (R2X = 0.366, Q2 = 0.315) (Figure 5A and 5B).

The collective comparison of the three diseases with the most important metabolites were arranged by their decreasing VIP values from the VIP-PLS-DA models in the following order: histidine, *O-*phosphocholine, glutamine, 2-oxoisocaproate, citrate, sarcosine, phenylalanine, ornithine, taurine, tryptophan, 2-hydroxyisovalerate, choline, lysine, glutamate, 3-methyl-2-oxovalerate, sn-glycero-3-phosphocholine, threonine, glucose, and proline. The unknowns resonance signals that were important in this comparison were unk_10, unk_4, unk_16, unk_11, unk_15, and unk_13. Among the metabolites, three based on loading plots were selected for each group. The following metabolites were important for the differentiation between the studied groups (Figure 5B): (1) AML group – phenylalanine, glutamate, and 2-oxoisocaproate; CLL group – choline, histidine, and *O-*phosphocholine; and (3) nHL – sarcosine, 2-hydroxyisovalerate, and sn-glycero-3-phosphocholine (Figure 5B).

Three separate chemometric models were calculated to determine accurate differences between the specific cancer groups. The metabolites that were statistically significant and present in the VIP-PLS-DA model after the first iteration are shown in Supplementary Table 2.

**Figure 6:**
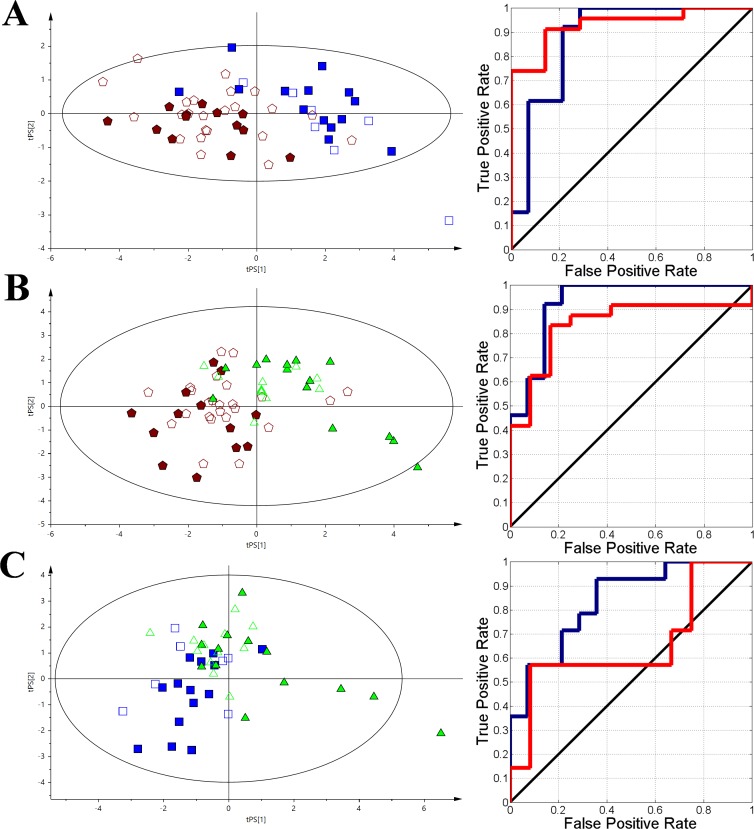
The VIP-PLS-DA model plots with the training (no fill) and test (fill) set samples and ROC curves The dark blue curve represents the training set and the red curve represents the test set. **(A)** - AML vs CLL; **(B)** - AML vs nHL, **(C)** - nHL vs CLL. Red pentagon – AML; Green triangle – nHL and Blue box – CLL.

Eight identified metabolites and 4 unknowns were used in the AML vs. CLL comparison (R^2^X = 0.459, Q^2^ = 0.383); phenylalanine, citrate, glutamine, *O-*phosphocholine, dimethyl sulfone, histidne, glutamate, choline, unk_11, unk_10, unk_7, and unk_18 are sorted in a descending order based on their VIP values (Supplementary Table 2 and Figure 6A). For the AML vs. nHL comparison (R^2^X = 0.530, Q^2^ = 0.575), glutamine, 2-oxoisocaproate, citrate, proline, sn-glycero-3-phosphocholine, 2-hydroxyisovalerate, unk_10, unk_11, and unk_16 (Supplementary Table 2 and Figure 6B) were selected, while glucose, *O-*phosphocholine, and tryptophan were used for the CLL vs. nHL comparison (R^2^X = 0.582, Q^2^ = 0.287) (Supplementary Table 2 and Figure 6C).

**Figure 7:**
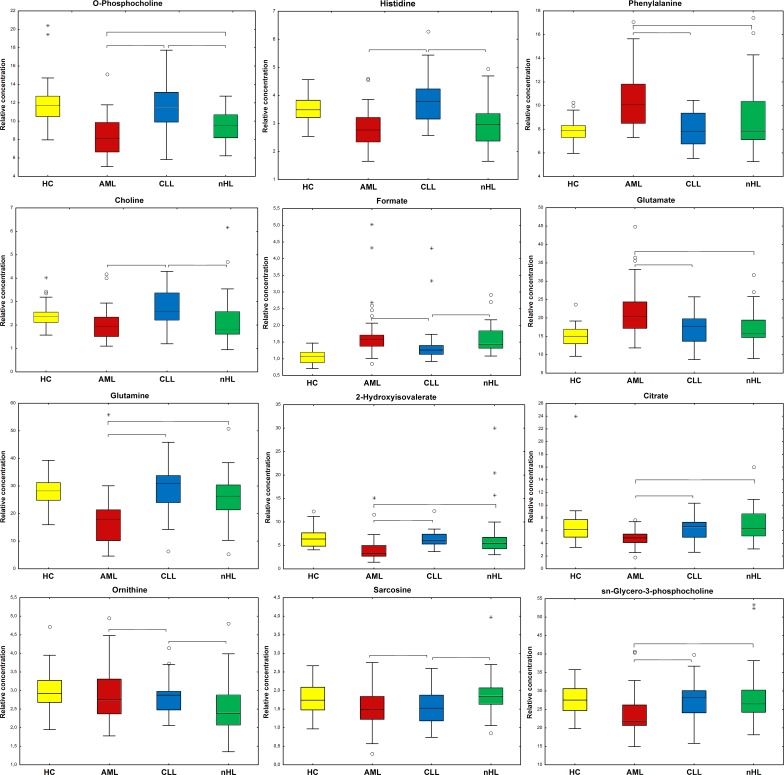
Boxplots for the identified statistically important metabolites (*p*<0.05) between the haematological cancer groups Braces show that the comparisons with the selected metabolites are statistically important. Whiskers - non-outlier min-max range; ^*^ - extreme; ° - outlier; bar - median; box - Q1-Q3 interquartile range;

Among the differentiating metabolites, compounds that were statistically significant in at least two comparisons between the haematological cancers groups in M-W or *T* tests (dependent on Shapiro Wilk test results) are presented in Figure 7.

### Pathway analysis

Pathway analysis was performed on the overall data, which contained the HC, AML, CLL, and nHL groups, and indicated the importance of the most changed biochemical pathways. The determined major changes are associated with the following pathways (Impact > 0.1; p Holm adjust <0.05): (1) D-glutamine and D-glutamate metabolism, (2) histidine metabolism, (3) alanine aspartate and glutamate metabolism, (4) arginine and proline metabolism, (5) glyoxylate and dicarboxylate metabolism, (6) aminoacyl-tRNA biosynthesis, (7) methane metabolism, (8) phenylalanine metabolism, (9) glycine serine and threonine metabolism, and (10) tryptophan metabolism.

## DISCUSSION

### Clinical significance

Haematological malignancies are a frequently diagnosed group of neoplasms and a significant cause of death induced by cancer [13, 14]. The successful treatment of these diseases is determined by early and accurate diagnosis. There are several cytologic, genetic and molecular tools used in diagnostic and monitoring hematological malignancies such as flowcytometry, FISH and PCR. Their sensitivity and clinical application however is heterogeneous.

In AML cytogenetic and molecular abnormalities are included in European Leukemia Net (ELN) prognostic classification [15]. The isolated mutation of NPM1 is related with favorable outcome whereas FLT3 ITD mutation is a poor prognosis factor. Recently, Gerstung et al. showed that more personal approach is needed and combination of several driver mutations play important role in clinical course of the disease [16]. Prognosis in CLL patients with del 17p by FISH remains poor.

Both in AML and CLL another important tools for measurement of minimal residual disease (MRD) is flow cytometry with high sensitivity. There is several studies showing that progression-free survival (PFS) and overall survival (OS) in patients MRD(-) is longer compared to MRD(+).

Most of methods mentioned need to be validated in prospective clinical trials. Moreover, introduction of new drugs - FLT3 inhibitors, BCR inhibitors, bcl2 inhibitors may abrogate prognostic value of cytogenetic and molecular abnormalities. Therefore more comprehensive approach including molecular and metabolomics tools may lead to personally tailoring cancer management.

Moreover, information about disease development could be valuable and supportive. The evaluation of changes in metabolite composition could be important for diagnosis, cancer stratification and as prognostic compounds with relevance for managing cancer therapies [17–20]. The objective of this study was to identify differences in metabolic changes between the healthy control group and the haematological malignancies groups with determination of the possible disturbances in the particular biochemical pathways. These findings might allow to better understand the nature of haematological disorders and enabling patient discrimination. Moreover, this approach could serve as supporting tool for earlier diagnoses and could lead to more suitable medical treatments.

According to acquired medical data (Supplementary Table 3) and clinical symptoms, AML is the most aggressive studied haematological malignancy. It causes the most visible changes to the serum metabolite profile ^1^H NMR spectrum. This is reflected in the multivariate data analysis and statistics tests (Tables 1 and Supplementary Table 2, Figures 3 and 6). In nHL group less differentiating results in all data analysis was obtained, in comparison to AML, which also supports the trend of groups aggressiveness (Supplementary Table 3). Moreover, the CLL group compared to healthy control group also follow this trend and highlights more subtle than in the both cases -AML or nHL group vs HC (Table 1).

Nevertheless, it is evident that some of the metabolites were essential for all three haematological cancers (Supplementary Table 2).

The detailed analysis of the average survival time (Supplementary Table 3) showed that the studied haematological malignancies could have been sorted as follows: the most aggressive is AML (avg. 2.74 ± 13.11 months), followed by nHL (avg. 7.35 ± 16.51 months), and ending with CLL (avg. 12.61 ± 13.40 months). This observation has been supported by the quality of the chemometric models, specifically the most aggressive cancer revealed the best separation from the HC group and the best chemometrics model parameters (Table 1).

The results obtained in our study compared well with previously reported data in the literature [17, 19]. The AML group is mostly consistent with overlapping in both studies statistically significant metabolites in the HC vs. AML comparison (Figure 3A and Supplementary Table 2), specifically the increase trends in phenylalanine in the AML group and the decrease in PC/GPC, choline, glutamine, and alanine were observed. The exception in our study (HC vs. AML) is the decrease in citric acid [17]. However, in another study based on GC-MS method, this metabolite was statistically significant and decreasing in the AML vs. HC comparison as in our study [19].

In literature there are not many scientific reports focused on investigating variations in low molecular weight compounds in nHL serum with general subgroups (Figure 3B and Supplementary Table 2) [21]. In this work all subgroups along with B lymphomas (with exclusion of CLL) were constituted one nHL group. Among the highlighted metabolites only phenylalanine matched with our study in the HC vs. nHL comparison [21]. In this study, this metabolite was increased for nHL but was not statistically significant. Moreover, only one literature reference is available and includes a metabolomics approach based on MALDI-TOF analysis of urine samples [22]. This publication highlights role of hypoxanthine as a main compound, which allows for nHL screening. The hypoxanthine levels in the nHL were significantly lower than in HC and permitted the differentiation of the selected groups. The finding obtained in our study shows highly elevated serum hypoxanthine (not statistically significant, VIP score > 0.8) in the nHL group, which is opposite to the results obtained by B.C. Yoo et al. [22]. The differences in these two studies [22] could be associated with differences in used type of biological material [23].

In the comparison between the CLL and control group, our results only partially overlap with the literature data (Figure 3C and Supplementary Table 2) [18]. Previously reported outcomes by MacIntyre et al. highlighted increased pyruvate, glutamate, and proline concentrations and decreased isoleucine level. Our results, which rely on statistically significant metabolites, only matched with one compound – glutamate. The other metabolites were not statistically significant in our study, and only proline followed the same trend. In contrast, isoleucine was slightly increased in the CLL group. The main difference between literature data and this study was inability to detect a pyruvate signal for quantification, pyruvate was reported as the most important metabolite for separation in the mentioned work [18]. In context of this data, observed higher glucose levels in the CLL group could be related to a potential comorbidity of the high-age patient group [18]. Analysis of the co-morbidities in our study showed that 5 out of 25 patients in the CLL group and 3 out of 28 in the HC group could potentially have disturbances in glucose levels (Supplementary Table 4). Therefore, in our opinion a consequence of the raised glucose level, which was statistically significant in the HC vs. CLL comparison, could be directly associated with development of CLL, though this requires further verification.

In spite of both being haematological malignancies, the AML and CLL groups significantly differed from one another [17, 18]. The alterations between the compounds in the different studies, which enabled their separation, could be caused by different disease stages or variability present in the taken control group. In case of CLL and nHL the separation was much less effective with coincides with the usual clinical presentation.

A recent study has reported that difficulties in cancer diagnosis could be due to the occurrence of co-morbidities in patients/healthy subjects and similar organism responses for different disease types [21]. It is known that additional factors related to immune system response may lead to more complex models that reflect the changes occurring in the cross-section of population [21]. Thus, it appears that important information from a diagnostic research could be obtained by comprehensive comparison of more than one disease with similar clinical symptoms or by having a more diverse control group.

### Biochemical insights into disease mechanism

To focus on the most important metabolites that allowed for the differentiation of studied groups, metabolites obtained from the complex model (HC vs. AML vs. CLL vs. nHL) were adopted, even when the model parameters were not as good as in the detailed comparisons (between two groups) (Supplementary Table 2). The group of metabolites was selected based on the K-W test (-log10(p) > 4.00) and VIP analysis (VIP value > 1.00) and includes O-phosphocholine, glutamine, phenylalanine, tryptophan, glutamate, histidine, formate, and 2-hydroxyisovalerate.

Throughout all the comparisons, *O*-phosphocholine appears to be one of most important metabolite, as it is widely considered to be a compound associated with the carcinogenic processes. *O*-phosphocholine is involved in glycerophospholipid metabolism, which is coupled to cancer cell metabolism and development [24]. Interestingly, this compound was considerably decreased in AML, displayed its lowest value among the haematological malignancies compared to the HC group. The second in *O*-phosphocholine level order in hetamological disorders is nHL and then CLL group with most similar value to HC (PC level HC>CLL>nHL>AML). The increased demand for glycerophospholipid metabolism compounds may be required for leukaemic cell proliferation. This suggests that the aggressiveness of the hematological diseases shows a decreasing trend among studied groups (AML>nHL>CLL) (Supplementary Table 3) [25, 26].

According to the list of the most influential metabolites examined in this study, glutamine was at the second position. In the literature, glutamine is reported as an essential compound for cancer development [27]. One of the most abundant uses of glutamine in biochemical pathways is its conversion to glutamate, which was also meaningful in our study. Glutamate is also transformed to α-ketoglutarate and is involved in powering the TCA cycle. It was important that glutamine (CLL> HC> NHL> AML) and glutamate (AML> NHL> CLL> HC) showed complementarity or a negative correlation, in the relative integrals, between each other (Figure 7, Supplementary Table 2). This may indicate an extensive intensification in the glutamine–glutamate biochemical transformation. These two compounds might be also associated with haematological cancer aggressiveness and use of these metabolites in TCA cycle amplification accompanied by other cancer development processes. In the literature, glutaminase inhibition highlights possible negative effects of cancer progression by interrupting the glutamine-glutamate pathway [28, 29]. Moreover, increased conversion of glutamine to glutamate may be essential for functioning of immune system cells [30].

Among amino acids, a decrease in tryptophan levels (HC> CLL> AML> nHL) could indicate increased tryptophan catabolism, which might be connected to degradation via the kynurenine pathway. This has been linked to local shutdown of the immune system response by indoleamine 2,3-dioxygenase (IDO) to promote malignant cell proliferation [31, 32, 33, 34]. Interestingly, the levels of the intermediate product - formate, is increased (Supplementary Table 2) and showed an opposing trend to tryptophan (AML>nHL>CLL>HC). This finding could support the changes, that occur throughout the mentioned biochemical pathway by accelerating tryptophan breakdown to kynurenine [35]. The expression of the IDO enzyme was reported in the literature for the AML group [32]. Interestingly, the nHL groups had the most intense changes in tryptophan levels. Non-Hodgkin's lymphomas are a heterogeneous group of lymphoid malignancies that are usually present in the lymph nodes, spleen and bone marrow. nHLs, most often among studied units, can infiltrate tissue and organs, which could lead to many local changes and favourable immunosuppressive microenvironments. Recent research shown that utilization of an IDO inhibitor can be a promising agent in the treatment of nHL patients [36].

Another remarkable low molecular weight compound from the amino acids group in the haematological malignancies was phenylalanine. Its relative integral increased in all three haematological disorders groups AML>nHL>CLL>HC and it could be related to protein breakdown. The BCAAs typically associated with this process are not statistically significant and do not cover the same order. Moreover, phenylalanine could amplify the requirement for fuelling tricarboxylic acid cycle for more aggressive haematological malignance through phenylalanine, tyrosine and tryptophan metabolism [37].

Our results showed that histidine negatively correlated with increased hematological diseases aggressiveness levels (CLL>HC>nHL>AML), which could be connected with inflammation processes [38, 39]. The histidine metabolism pathway also participates in glutamate biosynthesis. Thus, the increased transformation of histidine into glutamate may be cause of lower level in haematological patients.

The identification of 2-hydroxyisovalerate levels (nHL>CLL>HC>AML) in serum extracts of the haematological units was an unexpected observation, as it is most frequently assigned in urine as a potential indicator of lactic acidosis [40]. It was shown that lactic acidosis is a significant disturbance in the functioning of biochemical pathways in some haematological disorders studies [41, 42]. The main approach to determining the mentioned pathophysiological state was measuring lactate levels in blood. Compared to our results, elevated lactate levels were also observed in each unit, with the highest concentration visible in AML and nHL, which corresponds well with lactic acidic levels reported in the literature data [41, 42].

Considering the data originating from the general comparisons between the diseases (AML vs. CLL vs. nHL) (Figures 5D and 5E and Table 1) and beside the metabolites discussed earlier, we observed that 2-oxoisocaproate, citrate, sarcosine, ornithine, and taurine were the most important in second complex model.

2-oxoisocaproate (ketoleucine) is a short-chain keto acid, it along with other keto acids may be connected to kynurenine aminotransferase functioning as an amino group acceptor. However, its levels (AML>CLL>nHL>HC) were greatly increased in both of the leukaemia groups, though not in the lymphoma group (Supplementary Table 2) [43], which highlights the differences in the functioning of the selected haematological cancers.

The tricarboxylic acid cycle essential metabolite citrate (nHL>HC>CLL>AML) had the lowest relative integral in AML among investigated groups, indicating the ability of increased TCA cycle functioning to be related to the increased activation of carcinogenesis processes. Despite the different values in the CLL and nHL groups, neither displayed the considerable citrate reduction observed in the AML group. This suggests that differences in the levels of this compound could be associated with the increased energy demand correlated with the rapid development of AML [19].

The metabolism of sarcosine (nHL>HC>CLL>AML) leads to the rapid degradation to glycine, which demonstrates that serine and glycine metabolism may have an important impact on CLL and AML progression in contrast to nHL, it could also lead to betaine metabolism [44, 45].

Ornithine (HC>AML>CLL>nHL) is one of the main compounds in the urea cycle. The relative integral of this metabolite was lower in all three studied haematological cancers compared to the healthy control group. However, in the nHL samples, the ornithine levels differences were significantly higher than in the AML and CLL groups. Ornithine is mostly produced from arginine in the urea cycle, but could be also biosynthesized from proline and glutamine [46, 47]. In the literature, ornithine has been reported as an important agent for active T-cells. It can also lead to changes in biological pathways that involve ornithine usage and could be more prominent in nHL patients [46, 48].

The nHL group also had the lowest levels of taurine (CLL>HC>AML>nHL), and displayed significant differences from the other two studied groups. It has been reported that taurine could be early biomarker of tumour formation in breast cancer [49]. The literature data has reported that it is associated with a reduction of cancer development [50, 51]. Therefore, the decreased taurine levels in the nHL group correspond to its extensive utilization for reducing malignant cell proliferation [52].

The described changes can be responses amplified by tumour cells as well as global reactions by the entire organism to pathological conditions influenced by a variety of metabolites. The overall interpretation of the changes in the compound levels in serum samples could be difficult due to fact that metabolites within their biochemical pathways may intertwine and overlap during homeostasis disturbances and changes directly caused by cancer cell metabolism.

Summing up discrimination of the studied cancers was possible based on the detailed chemometric calculation between healthy control and cancer groups as well as between them. The found list of compounds may be key to understand the metabolite biochemical changes occurring in selected cancers, but also potentially allow for the classification and discrimination of haematological malignancies, based on differences in small molecular compounds composition.

Obtained models parameters were connected with the clinical symptoms assumptions and increased aggressiveness of the haematological units (AML survival time avg. 2.74 ± 13.11 months, nHL avg. 7.35 ± 16.51, CLL avg. 12.61 ± 13.40), and amplified by the changes in the biochemistry of metabolites. The discrimination based on the metabolomics approach was greater for the more aggressive units, which seems to be logical. This assumption was confirmed by the visible correlation between the quality and significance of the chemometric models compared to the average survival time in the groups (AML>nHL>CLL).

Hypothetically, the obtained results showed that this metabolomics approach could allow for the additional verification of diagnosis or even faster assignment of individuals to specific cancers in the future. However more accurate and detailed biochemistry studies are required to understand the changes in metabolism and their functions in the selected haematological cancers.

## MATERIALS AND METHODS

### Sample collection

Peripheral venous blood samples were drawn from all the participants after overnight fasting for at least 8 hours. Blood samples were collected using Sarstedt S-Monovette system serum tubes (Sarstedt AG & Co., Germany) that were centrifuged at 1000 x g for 15 minutes at 4°C. The serum samples serum were stored in 1,5 ml Eppendorf safe-lock tubes and maintained at −80°C until analysis.

The study group included 116 patients with an established diagnosis of one of three different haematological malignancies. There were 38 patients with acute myeloid leukaemia (AML), 26 patients with non-Hodgkin's lymphoma (nHL, CLL subgroup was excluded forming separate group) and 21 patients with chronic lymphocytic leukaemia (CLL). All the subjects were patients at the Haematology Clinic of Wroclaw Medical University in Poland. All recruited patients were in the active phase of the disease. All of the subjects had never been treated for their cancers. The serum samples were taken prior to the initiation of chemotherapy.

The control group consisted of 31 volunteers who were mainly recruited from familial doctors and from the Internal Diseases Clinic of Wroclaw Medical University in Poland. The volunteers were matched for age, sex, co-morbidities (arterial hypertension, diabetes, ischaemic heart disease, and hypercholesterolemia) and smoking habits to the haematological groups. Baseline demographic and medical characteristics for the haematological groups and controls are presented in Supplementary Tables 3, 4 & 5.

The study protocol was approved by the Bioethical Committee of Wroclaw Medical University (KB - 41/2011) and each subject gave written informed consent.

### Sample preparation for proton NMR spectroscopy

The collected serum samples were thawed at room temperature and vortexed. Then, 300 μl of serum was transferred to a new Eppendorf tube and mixed with 700 μl of cold methanol for protein precipitation. Next, the samples were homogenized (Qiagen, Tissuelyser LT) for 10 min at 50 Hz and then incubated for 20 min in −20°C. The homogenization step and incubation were then repeated. Subsequently, mixtures of serum-methanol were centrifuged for 30 min at 15 000 rpm at 4°C. Afterwards, 700 μl of supernatant was transferred to a new Eppendorf tube and then evaporated to dryness in a vacuum centrifuge (JWElectronic WP-03) for 5 h at 1500 rpm at 40°C. The dry precipitate was dissolved in 600 μl PBS (0.5 M, pH = 7.2, 20% D_2_O, and 330 μM TSP) and then 550 μl of the mixture was transferred to an NMR tube (SP, 5 mm ARMAR Chemicals). Samples were maintained at 4°C before the measurements were taken.

### ^1^H NMR measurements

The NMR spectra of the serum were recorded at 300 K using an Avance III spectrometer (Bruker, GmBH, Germany) operating at a proton frequency of 600.58 MHz. The NMR spectra of the were recorded by using a *cpmg1dpr* pulse sequence with water presaturation in Bruker notation. For each sample, 128 continuous scans were collected with a spin-echo delay of 400 μs; 80 loops; a relaxation delay of 3.5 s; an acquisition time of 2.73 s; a time domain of 64k; and a spectral width of 20.01 ppm. Two-dimensional NMR experiments (2D NMR) were recorded and processed for selected samples. Experiments performed included ^1^H−^1^H correlation spectroscopy (COSY), total correlation spectroscopy (TOCSY), and ^1^H−^13^C heteronuclear single quantum correlation (HSQC). For the metabolomics workflow, the 1D spectra were processed with a line broadening of 0.3 Hz, manually phased, baseline-corrected using the MestReNova software (Mestrelab Research v 11.0) and referenced to a TSP signal δ = 0.0 ppm for the serum samples. Methanol and water resonance signals were removed from the data matrix. All the spectra were normalized to a TSP resonance signal. The alignment of the resonance signals was completed via a correlation optimized warping algorithm (COW) [10] and the *icoshift* algorithm and implemented in MATLAB (v R2014a, Mathworks Inc.) [11].

### Pre-processing of variables for analysis

A total 50 metabolites and 18 unknown resonance signals from the serum sample ^1^H NMR spectra were assigned. The metabolite resonances were identified based on assignments published in the literature, the Chenomx software (v 8.2 Chenomx Inc.) and on-line databases (Biological Magnetic Resonance Data Bank (www.bmrb.wisc.edu) and Human Metabolome Data Base, (www.hmdb.ca). The NMR-measured metabolites were obtained as relative signal integrals of the non-overlapping resonance signals.

### Multivariate data analysis

Multivariate data analysis was performed using the SIMCA software (v 14.0, Umetrics). The sample order in the dataset was randomized. All the variables were scaled to unit variance (UV), and the samples for model calculation were split into two sample sets (training and test) based on the Kennard and Stone algorithm. The discriminant version of the Partial Least Squares regression (PLS-DA) with a default k-fold cross validation procedure was utilized to identify differences between the subgroups. To improve the obtained models, variable selection was conducted using VIP-plots with a confidence interval 0.95. Variables having VIP values below 0.8 were removed from the analysis. A single iteration was used to minimalize the overfitting models. New models were re-built based on selected variables, and their reliability were tested with a CV-ANOVA at a level of significance of p<0.05. The prediction performance of the VIP-PLS-DA models was estimated based on receiver operating characteristic (ROC) curves and area under curve (AUC) values. For this purpose, a *perfcurve* function from the Matlab statistical tool-box (Matlab v. R2014a, Mathworks, Inc.) was adopted. Specificity and sensitivity were determined according to sample class prediction using the 7-fold cross-validated predicted values from the fitted *Y*-predcv (implemented in SIMCA-14 software) for observations in the model.

### Pathway analysis

The MetaboAnalyst 3.0 platform with selected features was used for metabolite data analysis. Pathway Analysis was performed on a relative integral matrix with only identified metabolites. All the data were scaled with Auto Scaling; the Pathway Enrichment Analysis was set on Global Test and the Pathway Topology Analysis was set on Relative-betweenness Centrality [12].

### Statistical data analysis

The percent difference (PD) and relative standard deviation (RSD) for each assigned resonance signal were calculated. The percent difference was calculated based on the mean values of the metabolite's relative integral in each group. Data set with a relative integral were tested for the type of distribution based on the Shapiro-Wilk test. All assigned resonance signals were tested for statistical significance using an appropriate disturbance type test, either the Mann–Whitney–Wilcoxon or Student *t* test using STATISTICA 12 (Statsoft Inc.). The K-W test was calculated for limitation to most influential metabolites in discus section. K-W test was performed on the metaboanalyst platform for this purpose and all data were auto-scaled (http://www.metaboanalyst.ca) [12].

## SUPPLEMENTARY MATERIALS TABLES






