# Supplementary material for: Serum NMR metabolomics to differentiate haematologic malignancies
Source: Oncotarget. 2018 May 11;9(36):24414–27. doi: 10.18632/oncotarget.25311 (PMC5966245; doi:10.18632/oncotarget.25311)
Supplement: Supplementary file 1 [file oncotarget-09-24414-s001.pdf]

Serum NMR metabolomics to differentiate haematologic malignancies

SUPPLEMENTARY MATERIALS

Supplementary Table 1: List of metabolites used in the resonance signal assignment, the unidentified resonance signals and the chemical shifts for all the resonance signals used to develop the models. Signal clusters or partial resonance signals used in the calculations are shown in bold.

See Supplementary File 1

Supplementary Table 2: List of metabolites with their percent differences between the selected comparison and their relative standard deviations.

See Supplementary File 2

Supplementary Table 3: Average survival time in months of patients with the selected haematological malignancies (m/f)

	**AML (17/21)**		**nHL (11/15)**			**CLL (11/10)**		
**Survival time + SD**	**2.74 ± 13.11**		**7.35 ± 16.51**			**12.61 ± 13.40**		
**Risk factors**	Intermediate cytogenetic risk	High cytogenetic risk	IPI-low	IPI-intermediate	IPI-high	Standard-Risk	High-risk (deletion 17p)	
**n=**	23	15	4	14	8	17	4	
**Stage**	-			-		II	III	IV
**n=**	-			-		8	9	4

Supplementary Table 4: Detailed information about study participants' concomitant diseases

	(m/f)	HC (10/21)	AML (17/21)	nHL (11/15)	CLL (11/10)
	**Yes**	15 (1/14)	14 (4/10)	11 (6/5)	8 (5/3)
**Arterial hypertension**	**No**	15 (9/6)	18 (11/7)	13 (5/8)	8 (4/4)
	**No data**	1 (0/1)	6 (2/4)	2 (2/0)	5 (2/3)
	**Yes**	2 (0/2)	6 (1/5)	4 (2/2)	0
**Diabetes**	**No**	28 (10/18)	27 (15/13)	20 (9/11)	16 (8/8)
	**No data**	1 (0/1)	5 (1/4)	2 (2/0)	5 (3/2)
	**Yes**	1 (1/0)	9 (5/4)	3 (1/2)	2 (2/0)
**Smokers**	**No**	29 (9/20)	21 (9/12)	14 (6/8)	10 (3/7)
	**No data**	1 (0/1)	8 (2/6)	9 (6/3)	9 (6/3)
	**Yes**	1 (0/1)	7 (4/3)	3 (2/1)	0
**Coronary artery disease**	**No**	29 (10/19)	25 (12/13)	21 (9/12)	16 (8/8)
	**No data**	1 (0/1)	6 (1/5)	2 (2/0)	5 (3/2)
	**Yes**	0	0	0	0
**Hyperthyroidism**	**No**	30 (10/20)	32 (16/16)	24 (11/13)	16 (8/8)
	**No data**	1 (0/1)	6 (1/5)	2 (2/0)	5 (3/2)
	**Yes**	6 (0/6)	0	1 (1/0)	2 (2/0)
**Hypercholesterolemia**	**No**	24 (10/14)	28 (13/15)	18 (8/10)	15 (7/8)
	**No data**	1 (0/1)	10 (4/6)	7 (4/3)	4 (2/2)

Supplementary Table 5: Basic information from the study samples. Due to incomplete medical history from all the patients, the calculations for the included factors have been made using the number of samples shown in brackets

	Female	Male	All samples	HC	AML	nHL	CLL
**N (m/f) =**	67	49	116	31 (10/21)	38 (17/21)	26 (11/15)	21 (11/10)
**Age ± SD**	64.11 ± 13.71	63.95 ± 14.43	65.9 ± 14.02	63.54 ± 14.85	64.43 ± 14.90	65.65 ± 13.62	63.54 ± 12.37
**BMI ± SD**	27.95 ± 5.47 (49)	26.68 ± 5.16 (31)	27.46 ± 5.35 (80)	28.40 ± 5.06 (30)	27.3 ± 4.59 (25)	27.21 ± 6.65 (19)	24.19 ± 4.96 (6)
**WBC ± SD**	58.06 ± 155.47 (41)	38.67 ± 66.46 (35)	49.13 ± 122.43 (76)	-	48.02 ± 86.94 (32)	14.14 ± 17.49 (25)	97.04 ± 211.91 (19)
**RBC ± SD**	3.69 ± 0.86 (41)	3.71 ± 0.94 (35)	3.70 ± 0.89 (76)	-	3.04 ± 0.45 (32)	4.18 ± 0.9 (25)	4.18 ± 0.74 (19)
**Hb ± SD**	10.95 ± 2.16 (41)	11.24 ± 2.63 (35)	11.08 ± 2.38 (76)	-	9.39 ± 1.2 (32)	11.95 ± 2.38 (25)	12.79 ± 2.07 (19)
**PLT ± SD**	183.95 ± 139.69 (41)	131.74 ± 97.52 (35)	159.91 ± 124.11 (76)	-	84.31 ± 92.85 (32)	237.96 ± 132.93 (25)	184.53 ± 80.72 (19)
**CRP ± SD**	47.49 ± 60.17 (36)	40.54 ± 49.87 (32)	44.22 ± 55.26 (68)	-	59.12 ± 56.54 (31)	39.07 ± 57.49 (24)	18.18 ± 36.92 (13)
**LDH ± SD**	496.90 ± 541.37 (31)	502.52 ± 613.97 (31)	499.71 ± 574.05 (62)	-	634.43 ± 727.1 (23)	449.68 ± 499.71 (22)	382.18 ± 396.85 (17)
**Creatinine ± SD**	0.86 ± 0.32 (40)	1.09 ± 0.62 (35)	0.97 ± 0.5 (75)	-	0.9 ± 0.25 (32)	1.06 ± 0.76 (24)	0.97 ± 0.37 (19)
**eGFR ± SD**	80.21 ± 29.64 (40)	88.24 ± 41.73 (35)	83.95 ± 35.77 (75)	-	84.8 ± 28.88 (32)	85.03 ± 50.79 (24)	81.16 ± 22.72 (19)
**Urea ± SD**	36.73 ± 12.2 (30)	37.1 ± 16.02 (21)	36.88 ± 13.75 (51)	-	36.28 ± 12.56 (25)	38.79 ± 17.29 (14)	35.92 ± 12.44 (12)
**Uric acid ± SD**	5.46 ± 1.65 (39)	6.5 ± 2.3 (32)	5.93 ± 2.02 (71)	-	5.48 ± 1.86 (31)	6.47 ± 2.3 (24)	5.99 ± 1.8 (16)
**Fibrinogen ± SD**	3.65 ± 1.22 (32)	3.48 ± 1.47 (30)	3.57 ± 1.34 (62)	-	4.1 ± 1.24 (25)	3.26 ± 1.23 (20)	3.14 ± 1.4 (17)
**INR ± SD**	1.13 ± 0.32 (36)	1.1 ± 0.17 (30)	1.12 ± 0.26 (66)	-	1.26 ± 0.34 (27)	1.04 ± 0.13 (22)	0.99 ± 0.12 (17)
**D-dimer ± SD**	3.22 ± 6.62 (26)	2.44 ± 2.27 (22)	2.86 ± 5.08 (48)	-	3.97 ± 6.86 (24)	2.11 ± 1.63 (14)	1.25 ± 1.91 (10)
**APTT ± SD**	30.95 ± 10.49 (34)	30.33 ± 5.65 (26)	30.68 ± 8.67 (60)	-	32.87 ± 10.77 (26)	29.45 ± 5.57 (21)	28.28 ± 7.55 (13)
**GOT ± SD**	35.42 ± 27.89 (38)	28.35 ± 16.19 (34)	32.08 ± 23.23 (72)	-	36.27 ± 29.14 (30)	31.71 ± 18.96 (24)	25.61 ± 15.64 (18)
**GPT ± SD**	36.13 ± 34.23 (39)	26.06 ± 16.9 (34)	31.44 ± 27.84 (73)	-	43.42 ± 36.96 (31)	22.92 ± 16.48 (24)	22.17 ± 6.98 (18)
**Total bilirubin ± SD**	0.62 ± 0.36 (36)	1.12 ± 1.1 (31)	0.85 ± 0.83 (67)	-	0.93 ± 1.01 (30)	0.88 ± 0.8 (22)	0.65 ± 0.3 (15)
