# Supplementary material for: Serum NMR metabolomics to differentiate haematologic malignancies
Source: Oncotarget. 2018 May 11;9(36):24414–27. doi: 10.18632/oncotarget.25311 (PMC5966245; doi:10.18632/oncotarget.25311)
Supplement: Supplementary file 2 [file oncotarget-09-24414-s002.docx]

**Supplementary Table 1:** List of metabolites used in the resonance signal assignment, the unidentified resonance signals and the chemical shifts for all the resonance signals used to develop the models. Signal clusters or partial resonance signals used in the calculations are shown in bold.

| **Resonance signal assignment** | **^1^H δ = [ppm]** | **HMDB ID** |
| --- | --- | --- |
| 2-Hydroxybutyrate | **0.887 (t)**, 1.641 (m), 1.729 (m), 3.993 (dd) | HMDB00008 |
| 2-Hydroxyisobutyrate | **1.344 (s)** | HMDB00729 |
| 2-Hydroxyisovalerate | **0.822 (d)**, 0.953 (d), 2.006 (m), 3.839 (d) | HMDB00407 |
| 2-Methylglutarate | **1.058 (d)**, 1.590 (m), 1.748(m), 2.147(m), 2.244 (m) | HMDB00422 |
| 2-Oxoisocaproate | 0.925 (d), 2.082 (m), **2.599 (d)** | HMDB00695 |
| 3-Hydroxybutyrate | **1.188 (d)**, 2.296 (m), **2.393 (m)**, 4.142 (m) | HMDB00357 |
| 3-Methyl-2-oxovalerate | **0.882 (t)**, **1.086(d)**, 1.444 (m), 1.687 (m), 2.922(m) | HMDB00491 |
| Acetate | **1.906 (s)** | HMDB00042 |
| Alanine | **1.468 (d)**, 3.774 (q) | HMDB00161 |
| Alloisoleucine | **0.939 (m)**, 1.375 (m), 2.053 (m), 3.732 (d) | HMDB00557 |
| Betaine | 3.252 (s), **3.887 (s)** | HMDB00043 |
| Choline | **3.189 (s)**, 3.506 (dd), 4.056 (ddd) | HMDB00097 |
| Citrate | **2.523 (d)**, 2.673 (d) | HMDB00094 |
| Creatine | **3.024 (s), 3.918 (s)** | HMDB00064 |
| Creatinine | **3.033 (s), 4.046 (s)** | HMDB00562 |
| Dimethyl sulfone | **3.141 (s)** | HMDB04983 |
| Dimethylamine | **2.698 (s)** | HMDB00087 |
| Formate | **8.443 (s)** | HMDB00142 |
| Glucose | **3.237 (dd)**, **3.401 (m)**, **3.469 (m), 3.527 (dd)**, **3.711 (m)**, 3.823 (m), **3.889 (dd)**, 4.637 (d), **5.226 (d)** | HMDB00122 |
| Glutamate | 2.041 (m), 2.119 (m), **2.342 (m)**, 3.753 (dd) | HMDB00148 |
| Glutamine | 2.141 (m), **2.442 (m)**, 3.760 (t) | HMDB00641 |
| Glycerol | **3.552 (m), 3.644 (m)**, 3.776 (tt) | HMDB00131 |
| Glycine | **3.550 (s)** | HMDB00123 |
| Histidine | 3.15 - 3.34 (3.258) (m), 3.975 (dd), **7.068 (s)**, **7.832 (s)** | HMDB00479 |
| Hypoxanthine | **8.183 (s)**, 8.199 (s) | HMDB00157 |
| Isobutyrate | **1.050 (d),** 2.375 (m) | HMDB01873 |
| Isoleucine | **0.926 (t),** **0.997 (d)**, 1.248 (m), 1.459 (m), 1.969 (m), 3.661 (d) | HMDB00172 |
| Isovalerate | **0.898 (d)**, 1.939 (dq), 2.044 (d) | HMDB00718 |
| Lactate | **1.316 (d), 4.103 (q)** | HMDB00190 |
| Leucine | **0.948 (t)**, 1.698 (m), 3.730 (m) | HMDB00687 |
| Lysine | 1.463 (m), 1.715 (m), 1.892 (m), **3.018 (t)**, 3.749 (t) | HMDB00182 |
| N,N-Dimethylglycine | **2.914 (s)**, 3.711 (s) | HMDB00092 |
| *O-*Phosphocholine | **3.200 (s)**, 3.581 (m), 4.158 (dddd) | HMDB01565 |
| Ornithine | 1.737 (m), 1.821 (m), 1.934 (m), **3.045 (t)**, 3.777 (t) | HMDB00214 |
| Oxypurinol | **8.164 (s)** | HMDB00786 |
| Phenylalanine | 3.198 (m), 3.983 (dd), **7.320 (d)**, 7.366 (m), **7.418 (m)** | HMDB00159 |
| Proline | **1. 975 (m)**, 2.058 (m) 2.339 (m) 3.327 (dt), 3.412 (dt), 4.120 (dd) | HMDB00162 |
| Sarcosine | **2.741 (s)**, 3.599 (s) | HMDB00271 |
| sn-Glycero-3-phosphocholine | **3.213 (s)**, 3.637 (m), 3.907 (m), 4.313 (m) | HMDB00086 |
| Taurine | 3.261 (t), **3.419 (t)** | HMDB00251 |
| Threonine | 1.313 (d), **3.574 (d), 4.241(m)** | HMDB00167 |
| Tryptophan | 3.297 (dd), 3.475 (dd) 4.049 (dd), 7.190 (m), **7.274 (m)**, 7.317 (s), **7.534 (d)**, 7.725 (d) | HMDB00929 |
| Tyrosine | 3.043 (dd), 3.187 (dd), 3.934 (dd), **6.890 (m), 7.182 (m)** | HMDB00158 |
| Valine | **0.977 (d), 1.029 (d), 2.606 (m), 3.600 (d)** | HMDB00883 |
| Unknown_1 | **1.046 (s)** | - |
| Unknown_2 | **1.057 (s)** | - |
| Unknown_3 | **1.125 (d)** | - |
| Unknown_4 | **1.219 (d)** | - |
| Unknown_5 | **1.437 (d)** | - |
| Unknown_6 | **1.656 (m)** | - |
| Unknown_7 | **2.045 (m)** | - |
| Unknown_8 | **2.348 (m)** | - |
| Unknown_9 | **2.377 (s)** | - |
| Unknown_10 | **2.446 (s)** | - |
| Unknown_11 | **2.457 (s)** | - |
| Unknown_12 | **3.113 (s)** | - |
| Unknown_13 | **3.579 (s)** | - |
| Unknown_14 | **3.631 (s)** | - |
| Unknown_15 | **3.713 (s)** | - |
| Unknown_16 | **5.192 (d)** | - |
| Unknown_17 | **7.686 (s)** | - |
| Unknown_18 | **8.352 (s)** | - |
