# Supplementary material for: Serum NMR metabolomics to differentiate haematologic malignancies
Source: Oncotarget. 2018 May 11;9(36):24414–27. doi: 10.18632/oncotarget.25311 (PMC5966245; doi:10.18632/oncotarget.25311)
Supplement: Supplementary file 3 [file oncotarget-09-24414-s003.docx]

**Supplementary Table 2:** List of metabolites with their percent differences between the selected comparison and their relative standard deviations.

| **Metabolite** | **Percent difference** | | | | | | **Relative standard deviation [%]** | | | |
| --- | --- | --- | --- | --- | --- | --- | --- | --- | --- | --- |
|  | **HC vs. AML** | **HC vs. CLL** | **HC vs. nHL** | **AML vs. nHL** | **AML vs. CLL** | **nHL vs. CLL** | **HC** | **AML** | **CLL** | **nHL** |
| 2-Hydroxybutyrate | -36.04 ^a,b^ | -14.93 ^a^ | -47.40 ^a^ | -11.86 | 21.4 | 33.05 ^b^ | 24.21 | 53.58 | 25.24 | 81.61 |
| 2-Hydroxyisobutyrate | -7.23 ^b^ | -5.09 | -7.40 ^b^ | -0.17 | 2.14 | 2.31 | 27.82 | 41.21 | 67.13 | 55.73 |
| 2-Hydroxyisovalerate | 44 ^a,b^ | 2.03 | -10.9 | -54.25 ^a,b^ | -42.07 ^a^ | 12.92 | 30.23 | 63.90 | 29.37 | 82.92 |
| 2-Methylglutarate | -23.79 ^a,b^ | -9.06 ^b^ | -31.75 ^b^ | -8.11 | 14.81 | 22.85 ^b^ | 30.94 | 37.13 | 33.22 | 64.43 |
| 2-Oxoisocaproate | -44.8 ^a,b^ | -35.25 ^b^ | -8.42 | 36.72 ^a,b^ | 9.94 | -27.04 | 64.29 | 41.72 | 60.02 | 68.25 |
| 3-Hydroxybutyrate | -29.02 | -36.02 ^a^ | -67.51 | -40.48 | -7.19 | 33.53 | 37.22 | 80.51 | 57.65 | 198.51 |
| 3-Methyl-2-oxovalerate | -26.83 ^a,b^ | -30.63 ^a^ | -10.35 ^b^ | 16.6 ^b^ | -3.88 | -20.44 ^b^ | 41.39 | 43.70 | 45.06 | 48.07 |
| Acetate | -13.76 ^a,b^ | -13.51 ^b^ | -19.86 ^a^ | -6.15 | 0.25 | 6.4 | 16.92 | 25.72 | 29.27 | 38.70 |
| Alanine | 17.5 ^a^ | 12.32 ^a^ | 12.06 ^a^ | -5.46 | -5.2 | 0.26 | 16.62 | 22.09 | 25.78 | 25.43 |
| Alloisoleucine | -10.68 | -0.96 | 1.33 ^b^ | 12.01 ^a^ | 9.72 | -2.3 | 19.60 | 27.19 | 19.33 | 29.77 |
| Betaine | -13.06 ^a,b^ | -6.06 ^b^ | -7.51 ^b^ | 5.56 | 7.01 | 1.45 ^b^ | 14.43 | 18.89 | 18.17 | 16.17 |
| Choline | 17.3 ^a^ | -12.75 | 7.03 ^a^ | -10.3 | -29.88 ^a,b^ | -19.73 ^a^ | 20.96 | 33.82 | 32.23 | 51.43 |
| Citrate | 31.4 ^a^ | 4.21 ^b^ | -3.91 | -35.2 ^a,b^ | -27.28 ^a,b^ | 8.12 | 52.83 | 26.73 | 31.62 | 39.59 |
| Creatine | -9.14 | -16.59 ^b^ | -31.52 ^b^ | -22.54 ^b^ | -7.47 | 15.13 ^b^ | 38.00 | 46.85 | 40.24 | 63.69 |
| Creatinine | 0.32 | -8.06 | -0.39 | -0.7 | -8.38 | -7.68 | 13.27 | 22.23 | 25.92 | 24.74 |
| Dimethyl sulfone | 8.12 ^a^ | 5.08 | -5.16 | -13.27 ^b^ | -3.04 ^b^ | 10.24 | 25.45 | 90.18 | 37.61 | 33.39 |
| Dimethylamine | -46.95 ^a^ | -58.84 ^a,b^ | -51.75 ^a^ | -5.11 ^a^ | -12.77 | -7.67 | 70.00 | 65.07 | 74.23 | 62.30 |
| Formate | -48.79 ^a,b^ | -34.19 ^a,b^ | -40.73 ^a,b^ | 8.48 | 15.23 ^a^ | 6.78 ^a^ | 18.97 | 46.69 | 53.96 | 28.25 |
| Glucose | -11.49 | -13.27 ^a,b^ | 4.27 | 15.74 ^b^ | -1.79 | -17.52 ^a,b^ | 12.55 | 38.49 | 20.78 | 18.23 |
| Glutamate | -37.24 ^a,b^ | -14.18 ^a,b^ | -16.30 | 21.26 ^a^ | 23.36 ^a,b^ | 2.13 | 18.52 | 34.62 | 25.72 | 29.74 |
| Glutamine | 48.1 ^a^ | -0.65 | 11.01 | -37.59 ^a,b^ | -48.71 ^a,b^ | -11.65 | 18.66 | 54.78 | 30.25 | 36.56 |
| Glycerol | -4.92 | -2.67 ^b^ | 1.19 | 6.11 ^b^ | 2.25 | -3.86 | 20.31 | 27.63 | 30.88 | 17.23 |
| Glycine | -4.45 | -6.99 | 9.74 | 14.18 ^a^ | -2.54 | -16.71 | 22.32 | 26.24 | 49.86 | 21.09 |
| Histidine | 21.64 ^a,b^ | -9.62 | 15.19 ^a^ | -6.5 | -31.1 ^a,b^ | -24.72 ^a^ | 14.11 | 23.62 | 24.66 | 26.52 |
| Hypoxanthine | -4.16 | -28.33 | -31.84 ^b^ | -27.78 | -24.24 | 3.59 | 33.66 | 79.55 | 105.91 | 88.51 |
| Isobutyrate | -11.96 | 2.96 | -13.04 | -1.09 | 14.9 | 15.98 ^b^ | 18.27 | 39.16 | 28.38 | 37.17 |
| Isoleucine | 4.2 | -4.11 | -0.59 | -4.79 | -8.31 | -3.52 | 17.62 | 21.27 | 25.83 | 23.95 |
| Isovalerate | 4.83 | 2.15 | -4.87 | -9.69 | -2.68 | 7.02 | 21.06 | 35.74 | 32.69 | 31.99 |
| Lactate | -25.05 | -7.80 ^b^ | -26.31 | -1.29 | 17.33 | 18.61 | 23.08 | 81.11 | 57.14 | 77.95 |
| Leucine | -8.89 | 1.59 | -3.98 ^b^ | 4.91 | 10.47 | 5.57 ^b^ | 16.41 | 20.52 | 22.95 | 34.29 |
| Lysine | 2.07 | -5.6 | 5.62 | 3.55 | -7.67 | -11.21 ^a^ | 16.11 | 18.47 | 16.54 | 18.84 |
| N,N-Dimethylglycine | -15.29 | -16.62 ^a,b^ | -35.40 | -20.39 | -1.34 | 19.06 | 35.87 | 48.53 | 29.24 | 72.52 |
| *O-*Phosphocholine | 34.4 ^a,b^ | 2.33 | 24.45 ^a,b^ | -10.17 ^a^ | -32.14 ^a,b^ | -22.15 ^a,b^ | 21.79 | 25.56 | 24.92 | 16.93 |
| Ornithine | 3.11 | 5.14 | 17.11 ^a^ | 14.03 ^a^ | 2.03 | -12.00 ^a^ | 19.02 | 27.23 | 17.79 | 29.11 |
| Oxypurinol | -79.04 ^a^ | -89.19 ^a^ | -24.68 | 57.15 | -12.33 | -68.27 | 132.57 | 144.41 | 170.59 | 113.63 |
| Phenylalanine | -28.62 ^a,b^ | -1.27 ^b^ | -14.03 | 14.74 ^a^ | 27.38 ^a,b^ | 12.77 | 13.34 | 23.83 | 18.68 | 34.44 |
| Proline | 9.79 | -3.57 | -4.75 | -14.52 ^a,b^ | -13.35 | 1.18 | 26.26 | 19.68 | 26.61 | 26.54 |
| Sarcosine | 11.43 | 10.85 | -9.01 | -20.39 ^a^ | -0.59 | 19.81 ^a^ | 24.50 | 35.36 | 33.35 | 30.79 |
| sn-Glycero-3-phosphocholine | 16.09 ^a^ | 2.06 | -3.53 | -19.59 ^a,b^ | -14.05 ^a^ | 5.58 | 14.94 | 23.02 | 20.79 | 30.06 |
| Taurine | 3.53 | -1.17 | 14.48 ^a^ | 10.97 ^b^ | -4.7 | -15.64 ^a^ | 19.31 | 21.27 | 11.71 | 23.15 |
| Threonine | 3.69 | -6.6 | 4.98 | 1.29 | -10.28 | -11.57 | 15.61 | 18.23 | 21.51 | 16.32 |
| Tryptophan | 17.13 ^a,b^ | 5.34 ^b^ | 27.17 ^a^ | 10.16 | -11.82 | -21.91 ^a,b^ | 14.26 | 28.86 | 24.97 | 25.35 |
| Tyrosine | 0.92 | 4.22 | 6.42 | 5.5 | 3.3 | -2.2 | 17.43 | 21.05 | 29.09 | 26.82 |
| Valine | -1.45 | 5.5 | 7.08 | 8.52 | 6.94 | -1.59 | 14.63 | 17.44 | 21.72 | 25.54 |
| Unk_1 | 2.4 | 1.87 | -3.66 | -6.06 | -0.53 | 5.53 ^b^ | 18.71 | 31.89 | 23.48 | 26.70 |
| Unk_2 | 3.21 | -0.17 ^b^ | -9.50 | -12.69 | -3.37 | 9.33 ^b^ | 20.11 | 34.12 | 27.13 | 29.61 |
| Unk_3 | -64.92 ^a,b^ | -51.61 ^a,b^ | -49.28 | 16.99 | 14.52 | -2.49 | 74.61 | 98.49 | 65.27 | 140.89 |
| Unk_4 | 48.52 ^a,b^ | 3.04 | 22.69 ^a,b^ | -26.56 ^a^ | -45.65 ^a^ | -19.69 ^a^ | 26.47 | 49.00 | 31.58 | 36.93 |
| Unk_5 | -7.83 | -4.08 ^b^ | -3.97 | 3.87 | 3.75 | -0.11 | 14.51 | 43.85 | 33.66 | 46.19 |
| Unk_6 | -29.04 ^a,b^ | -21.97 ^a,b^ | -34.58 ^a^ | -5.68 | 7.19 | 12.86 ^b^ | 20.41 | 42.69 | 28.90 | 51.46 |
| Unk_7 | -37.02 ^a,b^ | -26.67 ^a,b^ | -40.92 ^a,b^ | -4.05 | 10.61 ^a,b^ | 14.64 ^a^ | 19.07 | 22.57 | 35.83 | 36.17 |
| Unk_8 | 11.47 | -5.52 | 1.45 | -10.02 | -16.96 | -6.98 | 41.72 | 36.33 | 33.29 | 31.63 |
| Unk_9 | -57.5 ^a,b^ | -47.39 ^a,b^ | -51.45 ^a^ | 6.54 ^a^ | 10.86 | 4.33 | 41.34 | 64.12 | 63.21 | 113.82 |
| Unk_10 | 24.11 ^a,b^ | -6.83 | -2.62 | -26.69 ^a,b^ | -30.81 ^a,b^ | -4.21 | 14.15 | 28.70 | 22.95 | 32.56 |
| Unk_11 | 25.99 ^a,b^ | -3.98 | -0.6 | -26.58 ^a,b^ | -29.89 ^a,b^ | -3.38 | 13.95 | 31.75 | 24.33 | 32.77 |
| Unk_12 | -27.79 ^a,b^ | -13.99 | -12.42 | 15.5 | 13.94 | -1.57 | 29.84 | 39.50 | 41.40 | 34.44 |
| Unk_13 | -7.45 | 4.27 | -2.31 | 5.14 | 11.71 | 6.58 | 21.10 | 18.74 | 24.96 | 19.96 |
| Unk_14 | -9.23 ^b^ | -5.63 | -10.08 | -0.85 | 3.61 | 4.45 | 23.82 | 28.06 | 25.57 | 26.26 |
| Unk_15 | -2.45 | -11.00 ^a^ | -4.65 ^a^ | -2.21 | -8.56 ^a^ | -6.36 ^a^ | 8.36 | 9.71 | 10.00 | 8.28 |
| Unk_16 | -53.15 ^a,b^ | -24.52 ^a,b^ | -16.11 | 37.85 ^a,b^ | 29.59 ^a^ | -8.5 | 27.39 | 38.52 | 45.17 | 47.74 |
| Unk_17 | -2.44 | -14.25 | -5.53 | 3.71 | 6.48 | -8.74 | 19.70 | 13.18 | 14.98 | 15.24 |
| Unk_18 | -67.28 ^a^ | 4.04 | 1.27 | 62.33 ^a^ | 54.33 ^a,b^ | 2.77 | 83.56 | 91.97 | 108.60 | 119.35 |

^a^ ‑ statistically important metabolites where (*p<0.05*). ^b^ - VIP-selected metabolites.
